# Results of antibiotic susceptibility testing do not influence clinical outcome in children with cystic fibrosis^☆^

**DOI:** 10.1016/j.jcf.2012.02.006

**Published:** 2012-07

**Authors:** M.N. Hurley, A.H. Amin Ariff, C. Bertenshaw, J. Bhatt, A.R. Smyth

**Affiliations:** a Division of Child Health, School of Clinical Sciences, University of Nottingham, Nottingham, NG7 2UH, UK; b Medical School, Faculty of Medicine and Health Sciences, University of Nottingham, Nottingham, NG7 2UH, UK; c Children and Young Persons CF Unit, Nottingham Children's Hospital, Queens Medical Centre, Nottingham, NG7 2UH, UK

**Keywords:** Cystic fibrosis, Antibiotic, Susceptibility testing, *Pseudomonas*

## Abstract

**Introduction:**

Patients with CF experience pulmonary exacerbations. These are often initially empirically treated with intravenous antibiotics, with antibiotic choice refined after susceptibility testing.

**Methods:**

We completed a 5-year retrospective review of children attending the Paediatric CF Unit, Nottingham. The respiratory sampling, antibiotic prescribing and susceptibility testing guidance were audited. Episodes were classified according to the concordance between the antibiotics prescribed and antibiotic susceptibility testing.

**Results:**

Of 52 patients who had previously isolated *Pseudomonas aeruginosa*, 103 antibiotic courses were commenced that coincided with an isolation of *P. aeruginosa*. *P. aeruginosa* was fully susceptible, partially susceptible or fully resistant on 33%, 44.7% or 16.5% of occasions respectively. The antibiotic prescriptions were never changed following antibiotic susceptibility testing. We found no association between change in FEV_1_ (p = 0.54), change in BMI (p = 0.12) or time to next exacerbation (p = 0.66) and concordance between antibiotic susceptibility and the antibiotics administered.

**Conclusion:**

This study contributes to mounting evidence questioning the utility of routine antibiotic susceptibility testing.

## 1 Introduction

The natural history of progression of lung disease in cystic fibrosis (CF) usually consists of a sequential acquisition of infecting organisms [2], the most significant acquisition being infection with *Pseudomonas aeruginosa*. While early infection with *P. aeruginosa*, may be eradicated by the expeditious use of antibiotic therapy [3–5], chronic infection with *P. aeruginosa* is accompanied by significant decline in clinical status, including lung function [6]. Such patients experience pulmonary exacerbations consisting of an increase in symptoms and reduction in lung function that are routinely treated with intravenous antibiotics [7].

Conventionally, antibiotic selection has been directed by the results of antibiotic susceptibility testing. However there is uncertainty regarding the states in which the organism exists within the CF lung and the antibiotic susceptibilities of the organism growing in planktonic, adherent and biofilm states vary significantly [8]. There is emerging evidence to suggest that the testing of antibiotic susceptibility against ceftazidime and tobramycin may not be associated with clinical response [9] and that when routine testing is reduced, short-term outcomes appear to be unaffected [10].

At the time of writing, the clinical guideline of Nottingham Children's Hospital Children and Young Persons CF Unit reflects the strategy of many units in the UK in recommending respiratory sampling at each clinical contact [11] and, at times of deterioration. When antibiotics are prescribed, routine antibiotic susceptibility testing of respiratory cultures guides the prescription. We wished to audit our practice against these guidelines and evaluate the outcome of our service.

## 2 Method

We completed a retrospective case-note audit of patients attending the Children and Young Persons CF Unit, Nottingham UK for each attendance between January 2005 and November 2010. We identified those patients who had chronic pulmonary infection with *P. aeruginosa* (as defined by greater than 50% of months with samples positive for *P. aeruginosa* over the last 12 months) [12].

The adherence to the respiratory sampling, antibiotic susceptibility testing and antibiotic prescribing guidance was audited. We determined the effect of adherence to the guidance upon outcome.

Our practice is to undertake respiratory sampling at the commencement of a treatment course of antibiotics, with the initial selection of antibiotics being either empirical or guided by the most recent culture result from the previous clinical contact. Antibiotics selection should then be refined once the results of susceptibility testing are reported. Antibiotic susceptibility testing is performed in our unit using disc diffusion testing against a standard panel of antibiotics — ceftazidime, ciprofloxacin, piptazobactam, meropenem, aztreonam, gentamicin, amikacin, tobramycin and colistin. The isolation of multiple strains of *P. aeruginosa* is identified by morphotype. The BSAC (British Society of Antimicrobial Chemotherapy) methods for antimicrobial susceptibility testing are used for interpretation [13].

We planned to identify episodes where a strain of *P. aeruginosa* was isolated from a patient with CF which was later reported to be resistant to the antibiotic(s) prescribed and where the antibiotics were not changed. We classified episodes according to the concordance between the antibiotics prescribed and antibiotic susceptibility profiles of the isolated organism. If the organism was ‘fully susceptible’ or ‘fully resistant’ to both antibiotics administered this was recorded (i.e. the organism was reported to be susceptible to both antibiotics empirically commenced — ‘fully susceptible’; or reported to be resistant to both antibiotics commenced — fully resistant). In cases where the organism was reported as susceptible to one antibiotic but resistant to the other in the combination, this was recorded as ‘partially susceptible’. In cases where more than one strain of *P. aeruginosa* was identified, the antibiotic profile was determined to be fully susceptible (or fully resistant) if the antibiotics prescribed matched (or otherwise) the susceptibility profile of the isolated organism. The combination of two strains of bacteria was considered to be ‘partially susceptible’ in all combinations other than the situation where the bacteria were fully susceptible or fully resistant (e.g. in the case of one strain being fully susceptible but the other partially susceptible, the combination was categorised as ‘partially susceptible’). Changes in FEV_1_, BMI (body mass index) during treatment and the time to next exacerbation were compared. Where BMI data was missing this was calculated from the last clinic height and the current weight. Two groups of analyses were performed; the first using the unit of analysis (UoA) as the first episode for each patient so that each patient would only contribute once, and for the second the unit of analysis was each exacerbation, to ensure that no data were lost. Differences between groups were tested using Kruskal–Wallis test.

## 3 Results

**Fig. 1:**
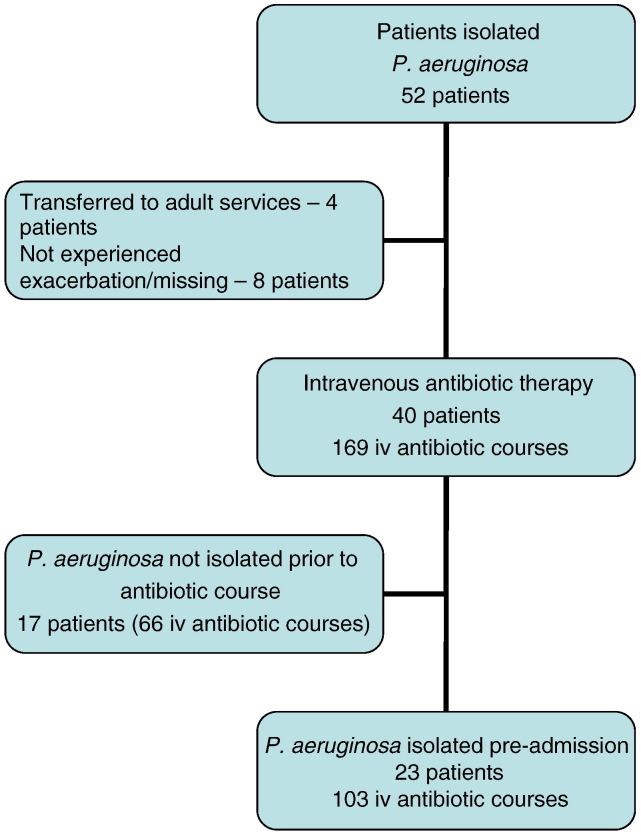
Flow diagram of patient/episode selection.

In the five year period, 52 patients had *P. aeruginosa* isolated at least once, of whom 4 had been transferred to adult services and 8 patients had not experienced a pulmonary exacerbation or their notes were ‘missing’. The remaining 40 patients had received 306 antibiotic courses combined, of which 169 (55.2%) were given intravenously. Of these, 23 patients had received 103 courses of intravenous antibiotic therapy that followed an isolation of *P. aeruginosa* either on admission or at a clinic attendance that prompted admission. This group forms the basis of the further analysis. (Fig. 1).

Of the episodes where *P. aeruginosa* was isolated and treated with intravenous antibiotics, the average age at first intravenous antibiotic course in the study period was 9 years (range 1–14 years) with patients receiving an average of 4 intravenous courses (range 1–17) in the period of interest.

Reports for antibiotic susceptibility testing were available for 97 (94.2%) episodes. In no case was the antibiotic prescription changed upon receipt of the antibiotic susceptibility report. Of the 103 episodes, the organism was found to be fully susceptible to the antibiotics administered on 34 (33%), fully resistant on 17 (16.5%) and partially susceptible on 46 (44.7%) of occasions. On three occasions the antibiotic prescription was changed mid-way through an intravenous treatment course. On two occasions this was due to a poor clinical response, however the antibiotic change did not alter the concordance categorisation. On a third occasion a family member wished the antibiotics to be changed and again, the antibiotic was changed to another to which the organism was similarly resistant.

**Fig. 2:**
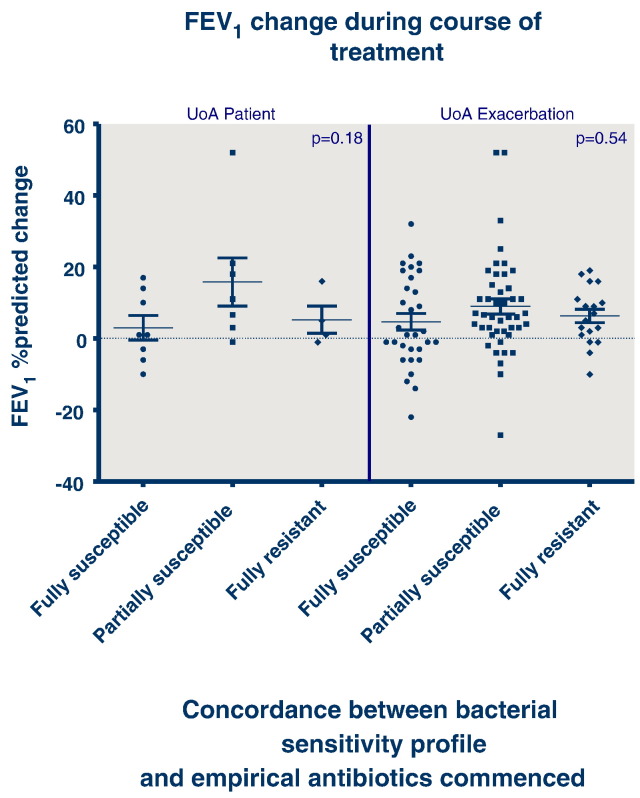
FEV1 change over the duration of an antibiotic treatment course with categorisation of antibiotic concordance (UoA — unit of analysis).

**Fig. 3:**
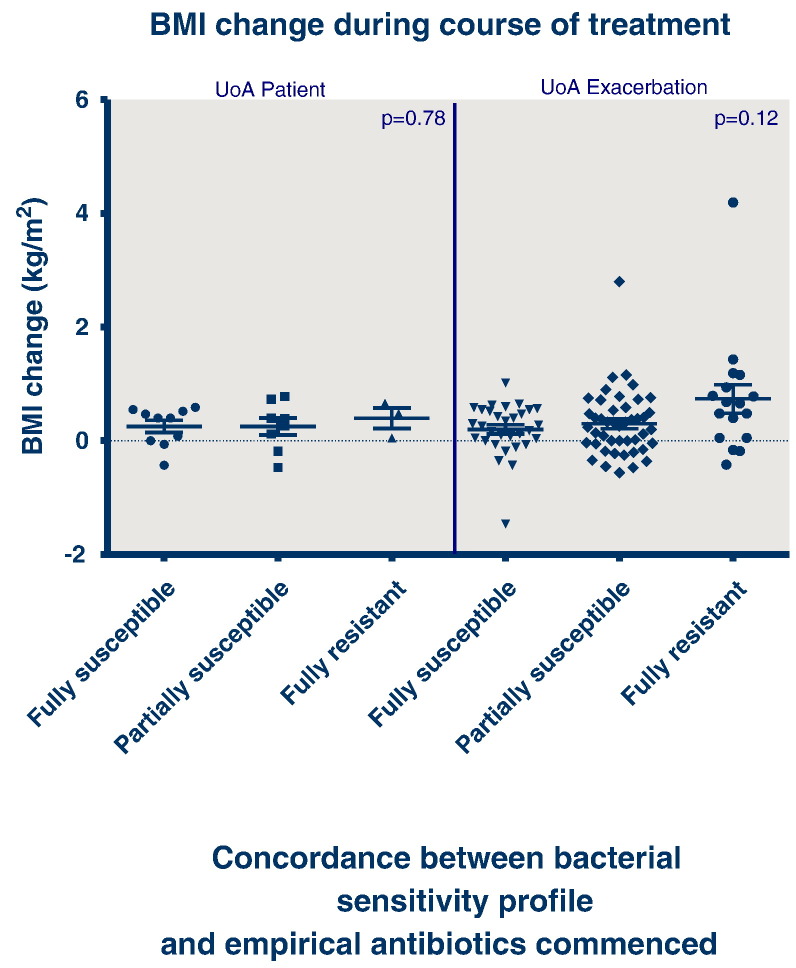
BMI change over the duration of an antibiotic treatment course with categorisation of antibiotic concordance (UoA — unit of analysis).

**Fig. 4:**
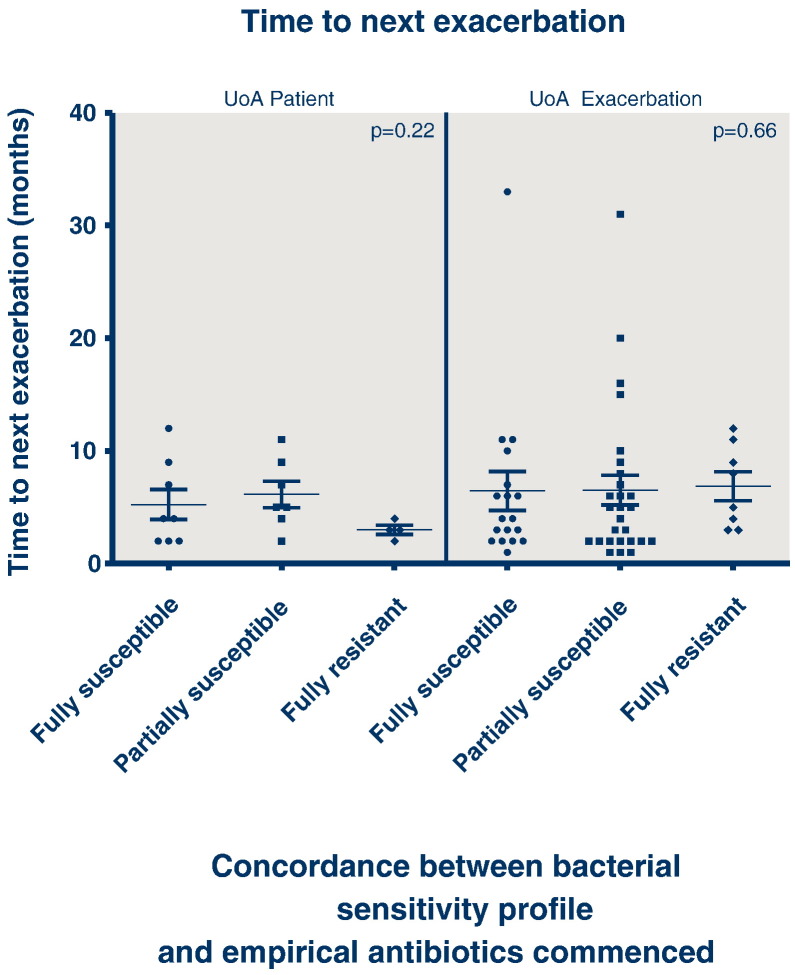
Time to next exacerbation/course of iv antibiotics categorised by antibiotic concordance (UoA — unit of analysis).

Considering the outcomes of FEV_1_ (% predicted) (Fig. 2), BMI (Fig. 3) or time to next exacerbation/next intravenous treatment course (Fig. 4), there was no association between outcome and concordance between antibiotic susceptibility profile and the antibiotics administered.

Analysing all 103 episodes, the outcome in terms of change in FEV_1_ percent predicted was not associated with the concordance between sensitivity profile and the antibiotics prescribed (p = 0.5353), neither was outcome dependent upon antibiotic concordance for change in BMI (p = 0.1202), or time to next exacerbation of intravenous antibiotic course (p = 0.6603).

Analysing the data using the patient as the unit of analysis so that each patient contributes only their first treatment course to the data set, neither FEV_1_ (p = 0.1759), BMI (p = 0.7785) or time to next exacerbation or intravenous antibiotic course (p = 0.2239) was associated with antibiotic susceptibility concordance.

## 4 Discussion

We found that our practice adhered closely to the guideline recommending respiratory sampling at each clinical contact and that antibiotic susceptibility testing was performed in 94.2% of cases. However on no occasion was the empirical antibiotic changed upon receipt of the antibiotic susceptibility profile. We found that, despite 66% of antibiotic courses consisting of antibiotics to which the organism was not fully susceptible, the antibiotic concordance was not associated with clinical outcome in terms of lung function (FEV_1_), BMI or time to the next exacerbation.

A similar study consisting of a retrospective analysis of patient data from the control group of a randomised trial of inhaled tobramycin, restricted the prescription of intravenous antibiotics to ceftazidime and tobramycin. They found no correlation between antibiotic susceptibility to tobramycin and ceftazidime and clinical outcome [9]. In their practice in an adult CF unit, Etherington et al. [10] rationalised antibiotic susceptibility testing to be performed only at the onset of antibiotic treatment, upon suspicion of clinical treatment failure or routinely if not tested in the previous three months [10]. They analysed the response to treatment before and after the change in practice and found no significant difference.

The antibiotic susceptibility reports in these studies detail susceptibility profiles for antibiotics in isolation. In order to answer the question of whether combination antibiotic susceptibility testing conferred a clinical benefit, a randomised trial compared combination susceptibility testing with conventional testing. There was no significant difference in clinical or bacteriological outcome [14].

In order to more closely reflect the anticipated mode of growth of the organism within the CF lung, attempts have been made to consider antibiotic regimens based on biofilm susceptibilities. Generating simulated antibiotic regimens based on biofilm and conventional susceptibility testing yields different antibiotic selections that on some occasions were contradictory [15]. The results of clinical trials evaluating biofilm susceptibility-directed therapy are awaited.

Our report is likely to represent a significant proportion of clinical practice in the UK and reflects the ‘real world situation’. Not only was the response to all antibiotic selections included, but we were also able to determine the effect for patients who receive intravenous antibiotics at regular intervals, irrespective of whether they would meet criteria of an infective exacerbation. We are also the first to report such findings in the paediatric population.

Our findings are limited by the small number of patients who contribute to the data set. However, these patients represent a wide spectrum of severity of disease and the corresponding analysis at the patient level, serves to support our findings and reduce the likelihood that we have encountered a unit of analysis error.

The concern regarding the applicability of conventional antibiotic susceptibility continues to increase and further questions arise as a result. The most likely explanation for the poor association between susceptibility and response is that the mode of growth of the organism is difficult to represent *in vitro*. In addition, inconsistencies in the diagnosis of pulmonary exacerbations [16] and uncertainty about under which circumstances and for what duration antibiotics should be used [17] add to the lack of confidence in our current antibiotic management of infection in CF.

The effective management of pulmonary exacerbations is critical to the continued improvements in CF care, and is an area in which we currently fall short. Twenty-five percent of patients experiencing an exacerbation do not recover their baseline lung function after treatment [18] leading to the relentless deterioration in clinical state over time. The administration of intravenous antibiotics is also associated with toxicity, selecting for resistant strains and significant costs both for patients and healthcare systems. The judicious use of antibiotics is required however we currently are unable to more effectively target the causative organism. A focus for research is the search for a valid *in vitro* model of the *in vivo* condition and further understanding of how the organism behaves in the clinical setting. In the meantime the mounting evidence questioning the utility of routine susceptibility testing suggests that a more refined approach should be considered.
