# Supplementary material for: Results of antibiotic susceptibility testing do not influence clinical outcome in children with cystic fibrosis
Source: J Cyst Fibros. 2012 Jul;11(4):288–92. doi: 10.1016/j.jcf.2012.02.006 (PMC3382712; doi:10.1016/j.jcf.2012.02.006)
Supplement: Supplementary file 1 [file mmc1.doc]

***The Journal of Cystic Fibrosis***

Review File

Results of Antibiotic Susceptibility Testing do not Influence Clinical Outcome in Children with

Cystic Fibrosis

Matthew N Hurley, Alifa H Amin Ariff; Carol Bertenshaw;

Jayesh Bhatt; Alan R Smyth,

**Review timeline:**

Submission date: 17 August 2011

Editorial Decision: 23 January 2012

Revision received: 21 February 2012

Additional Correspondence: 22 February 2012

Accepted: 24 February 2012

**Transaction Report:**

(Note: With the exception of the correction of typographical or spelling errors that could be a source of ambiguity, letters and reports are not edited. The original formatting of letters and referee reports may not be reflected in this compilation.)

***1st Editorial Decision***

Reviewers have now commented on your paper. You will see that they are advising that you revise your manuscript. If you are prepared to undertake the work required, I would be pleased to reconsider my decision.

Finally, I would appreciate if you could submit your revised paper by Feb 22, 2012.

Yours sincerely

Editor-in-Chief

Journal of Cystic Fibrosis

**REFEREE REPORTS**

**Referee 1 (Remarks to the Author):**

Authors in this study try to establish a correlation between antibiotic susceptibility testing and clinical outcome in cystic fibrosis patients. This type of study has been previously performed and findings fail to establish that correlation. This idea is quoted by the authors in the introduction with appropriate references.  Nevertheless these studies were mainly performed with adult patients.  The study merits publication but requires clarifications in the definitions and microbiology methods.

Comments.
1. The term "susceptible" should be used instead "sensitive". The former is commonly used by breakpoint committees and it is used in clinical category definition. Moreover, definitions are not clear. The following terms should be clearly defined in the Material and Methods section: fully sensitive and fully resistant and differences with sensitive or resistant.    
2. Define in Material and Methods which methods was used for susceptibility testing (disk diffusion, microdilution, …). Moreover, which were the criteria followed for interpretation ot susceptibility testing results (BSAC, CLSI, EUCAST…)
3. Which antibiotics were recorded?
4. Were co-colonization recorder? And bacterial load?  
5. Which was the criteria used to identify more than one strain of P. aeruginosa in bacterial cultures (morphotype, typing methods, …)
6. Figures. Clarify "UoA"

***1st Revision - authors' response***

We are grateful for the opportunity to improve our paper based on the comments of the peer reviewer.

Please find enclosed our revision and our responses to the peer review.

1. The term "susceptible" should be used instead "sensitive". The former is commonly used by breakpoint committees and it is used in clinical category definition. Moreover, definitions are not clear. The following terms should be clearly defined in the Material and Methods section: fully sensitive and fully resistant and differences with sensitive or resistant.

Response: Agree and changed. The terms fully susceptible and partially susceptible are now used.

The explanation of terms in the method has been supplemented for clarity. If the organism was ‘fully susceptible’ or ‘fully resistant’ to both antibiotics administered this was recorded (i.e. the organism was reported to be susceptible to *both* antibiotics empirically commenced – ‘fully susceptible’; or reported to be resistant to *both* antibiotics commenced – fully resistant).

2. Define in Material and Methods which methods was used for susceptibility testing (disk diffusion, microdilution, .). Moreover, which were the criteria followed for interpretation of susceptibility testing results (BSAC, CLSI, EUCAST.)

Response: The original submitted version of our paper identified the BSAC methods as being used. We have added the detail regarding disc diffusion testing and the panel of antibiotics which are routinely tested.

3. Which antibiotics were recorded?

Response:This detail has been added with comment above.

4. Were co-colonization recorder? And bacterial load?

Response: See below. We do not assess bacterial load.

5. Which was the criteria used to identify more than one strain of P. aeruginosa in bacterial cultures (morphotype, typing methods).

Response: The sentence has been added to clarify – The isolation of multiple strains of *P. aeruginosa* is identified by morphotype

6. Figures. Clarify "UoA"

Response: UoA is an abbreviation of ‘unit of analysis’ as first introduced in the methods. The abbreviation has been explained in the legend for the graphs for clarity.

In re-reading the paper in response to the peer reviewers comments we have amended a number errors/made changes:

Line number of original submission

Error/reason for change Amendment

Page 6 line 32: We have been informed that the guidelines will no longer be publicly available online Web address removed

Page 7 line 55-56: The references for the figures are incorrect

Figure numbers corrected: FEV1 figure 2 etc.

Page 9 line 12: The statement regarding antibiotic assumption of efficacy was misinterpreted from the

CFF Pulmonary guidelines

Sentence deleted.

Figure 1 A: typographical error was inserted in the chart Number of patients not isolating

PA prior to antibiotic course should be 17 not 20.

References: Incomplete reference cited for Cochrane review

Reference updated.

***2nd Additional Correspondence***

Thank you for submitting your revised manuscript for our consideration. We have now heard back from the referees who have reevaluated the study (see comments below), and I am pleased to inform you that both of them now consider the study suitable for publication. We shall therefore be happy to proceed with its acceptance and production.

Yours sincerely

Editor-in-Chief

Journal of Cystic Fibrosis
